# Population structure and genetic diversity analyses of common bean germplasm collections of East and Southern Africa using morphological traits and high-density SNP markers

**DOI:** 10.1371/journal.pone.0243238

**Published:** 2020-12-18

**Authors:** Wilson Nkhata, Hussein Shimelis, Rob Melis, Rowland Chirwa, Tenyson Mzengeza, Isack Mathew, Admire Shayanowako

**Affiliations:** 1 African Centre for Crop Improvement, School of Agricultural, Earth and Environmental Sciences, University of KwaZulu-Natal, Pietermaritzburg, South Africa; 2 International Centre for Tropical Agriculture, Chitedze Agricultural Research Station, Lilongwe, Malawi; 3 Department of Agricultural Research Service, Chitedze Agricultural Research Station, Lilongwe, Malawi; National Bureau of Plant Genetic Resources, INDIA

## Abstract

Knowledge of genetic diversity in plant germplasm and the relationship between genetic factors and phenotypic expression is vital for crop improvement. This study's objectives were to understand the extent of genetic diversity and population structure in 60 common bean genotypes from East and Southern Africa. The common bean genotypes exhibited significant (p<0.05) levels of variability for traits such as days to flowering (DTF), days to maturity (DTM), number of pods per plant (NPP), number of seeds per pod (NSP), and grain yield per hectare in kilograms (GYD). About 47.82 per cent of the variation among the genotypes was explained by seven principal components (PC) associated with the following agronomic traits: NPP, NFF (nodes to first flower), DTF, GH (growth habit) and GYD. The SNP markers revealed mean gene diversity and polymorphic information content values of 0.38 and 0.25, respectively, which suggested the presence of considerable genetic variation among the assessed genotypes. Analysis of molecular variance showed that 51% of the genetic variation were between the gene pools, while 49% of the variation were within the gene pools. The genotypes were delineated into two distinct groups through the population structure, cluster and phylogenetic analyses. Genetically divergent genotypes such as DRK57, MW3915, NUA59, and VTTT924/4-4 with high yield and agronomic potential were identified, which may be useful for common bean improvement.

## Introduction

Common bean (*Phaseolus vulgaris* L. 2n = 2x = 22) is one of the principal grain legume in the world. In Africa, itis the most important source of dietary protein [1] and the third most important source of calories after maize (*Zea mays* L.) and cassava (*Manihot esculenta* Crantz), serving millions of low-income households [2]. The global production of common bean is nearly 12 million tons per annum. The East and Southern Africa regions produces about 2.5 million tons per annum [3]. Approximately 40 per cent of Africa’s production is marketed for about 450 million US dollars [4], and small holder farmers account for the bulk of the cultivated crop.

The average yield for common bean in Southern Africa is very low (<200 kgha^-1^) compared to the global average of 2,000 kgha^-1^ [5, 6]. The low productivity of common bean is attributable to an array of biotic and abiotic constraints. Therefore, there is a need to develop high yielding and stress-tolerant cultivars to improve productivity. The successful development and deployment of improved cultivars depend upon available genetic diversity and appropriate breeding strategies.

Genetic variation in the common bean is derived from two major gene pools, which are primarily differentiated by their centres of diversity. These gene pools are from the Mesoamerica centre of diversity that extends from Colombia to Northern Mexico and the Andes covering the area from North-Western Argentina to Southern Peru [7]. The differences between these two gene pools have been reported through several genetic and morphological studies in selected agro-ecologies [8, 9]. The accessions of Andean origin are described as large-seeded, while the Mesoamerican accessions are small-seeded types [8].

After the introduction of the common bean in the 16^th^ and 17^th^ centuries in Africa [10], the crop has undergone natural and human selection pressure resulting in genetic divergence compared with the original Andean and Mesoamerican gene pools. Gene flow among different gene pools and or within races through natural cross-pollination has resulted in the diversification of landraces in Southern Africa. Following years of selection and adaptation, the landraces found in Southern Africa have evolved as distinct types with distinguishable morphological features. The East and Southern African regions are now recognized as secondary centres of genetic diversity for common bean [11]. Thus, germplasm from the East and Southern African regions complement the original gene pools and provide essential genetic variation for breeding. Assessing the genetic diversity among genotypes collected from different geographical locations is important to understand genetic composition and gene loci differentiation in common bean for cultivar development [12].

Knowledge of genetic diversity in plant germplasm and the interrelationship between genetic markers and phenotypic expression is vital for crop improvement. This will enhance efficiency during germplasm management, selection, and cultivar development [13, 14]. Diversity studies in common bean utilize both morphological and molecular markers [15–17]. However, morphological markers are highly affected by environmental variance, which reduces selection efficiency during cultivar development. The use of molecular markers has gained prominence for genetic diversity assessment because they are not affected by environmental conditions. Their determination is mostly automated, which reduces human experimental errors. Molecular markers, including random amplified polymorphism DNA (RAPD), simple sequence repeats (SSR), amplified fragment length polymorphism (AFLP), and single nucleotide polymorphism (SNP) have been used widely in genetic studies on common bean [8, 18–20]. Recently, SNP markers have gained prominence in genetic diversity studies in common bean [17, 21]. Their prominence has increased because SNP markers are more abundant across the genome, highly reproducible, and can be easily used in automated systems [17, 21]. The advent of the next-generation sequencing platform has enabled the discovery of more than a million SNP markers in common bean. These SNP markers have been used to develop linkage maps, mapping of quantitative trait loci (QTL), map-based gene cloning, marker-assisted selection, and exploration of genetic diversity [22–25]. Common bean breeding programs in East and Southern Africa can benefit from assessing genetic diversity in different germplasm using SNP markers. This will enable effective genetic management and accelerated genetic advancement for cultivar development.

National and international germplasm exchange and informal trade have resulted in considerable gene flow among germplasm collections between East and Southern African countries over the last 30 years [26]. Although both locally available germplasm and introductions have been used as cultivars in East and Southern Africa [21], the functional genetic diversity among these genetic resources is yet to be fully explored for efficient breeding. In Africa, the characterization of crop genetic resources has been mostly focused on phenotypic evaluation with limited use of genomic tools. Few studies assessed genetic diversity and deduced population structure based on sources of collection, races, and gene pools [12, 27]. Some studies sought to evaluate gene flow among different populations using molecular markers such as simple sequence repeats (SSR) [11, 28]. Assessing gene flow among germplasm collections from diverse geographical locations has been a proxy for estimating potential genetic diversity among common bean germplasm for breeding. The evolution of landraces, cultivars, and lines from different locations due to differences in selection pressure results in genetic divergence from the original Andean and Mesoamerican gene pools. Hence, a large proportion of common bean genetic resources remains uncharacterized and under-utilized [29]. For instance, genetic variation for bean fly resistance has not been widely assessed, and genetic studies on bean fly resistance within East and Southern African common bean germplasm collections are scarce despite their importance as sources of genetic diversity. In addition, the genetic basis for adaptive traits against stresses such as bean fly infestation is still to be elucidated [30]. This is partly attributable to phenotyping difficulties for bean fly resistance and a lack of systematic and efficient screening procedures [29]. Thus, there is also a need to improve phenotyping procedures to generate complementary phenotypic data for genetic diversity studies. Therefore, the objectives of this study were to understand the extent of genetic diversity and population structure in 60 common bean germplasm collections from East and Southern Africa.

## Materials and methods

### Germplasm

The germplasm used in this study consisted of 60 common bean genotypes collected from the Malawi Department of Agricultural Research Services (DARS/Malawi) and the International Centre for Tropical Agriculture (CIAT), (Table 1). Forty-five genotypes were obtained from DARS, Malawi, which included conserved landraces and released cultivars.

**Table 1:** Entry code, name, and description of 60 common bean genotypes used in the study.

Entry	Name/designation	Gene pool	Source	Description	Seed colour
E28	A286	Andean	CIAT	Breeding line	Carioca
E30	SER265	Mesoamerican	CIAT	Breeding line	Red
E42	SER267	Mesoamerican	CIAT	Breeding line	Red
E51	CAL143	Andean	CIAT	Breeding line	Red mottled
E69	G11982	Andean	CIAT	Breeding line	Red speckled
E74	SER124	Mesoamerican	CIAT	Breeding line	Red
E78	CAL96	Andean	CIAT	Breeding line	Red mottled
E89	A344	Mesoamerican	CIAT	Breeding line	Carioca
E93	MW3954	Andean	DARS/MW	Breeding line	Red mottled
E2	MW3991	Andean	DARS/MW	Landrace	Cream
E9	MW3983	Andean	DARS/MW	Landrace	Cream
E10	MW3969	Andean	DARS/MW	Landrace	Red
E11	MW3955	Andean	DARS/MW	Landrace	Red
E12	MW3928	Andean	DARS/MW	Landrace	Red speckled
E14	MW3927	Andean	DARS/MW	Landrace	Dark red
E16	Nasaka	Andean	DARS/MW	Landrace	Khaki
E21	MW4011	Mesoamerican	DARS/MW	Landrace	Cream
E22	MW4012	Mesoamerican	DARS/MW	Landrace	Light speckled
E23	MW3964	Andean	DARS/MW	Landrace	Red
E24	MW3929D	Andean	DARS/MW	Landrace	Cream
E26	MW3929C	Andean	DARS/MW	Landrace	Light speckled
E33	MW4023	Andean	DARS/MW	Landrace	Purple
E34	MW4018	Andean	DARS/MW	Landrace	Cream
E35	MW3997	Andean	DARS/MW	Landrace	Dark red
E36	MW3960	Andean	DARS/MW	Landrace	Red mottled
E38	MW4020	Andean	DARS/MW	Landrace	Cream
E40	MW3966	Andean	DARS/MW	Landrace	Red mottled
E44	MW3241	Mesoamerican	DARS/MW	Landrace	Dark red
E45	MW4090	Andean	DARS/MW	Landrace	Red
E46	MW3946	Andean	DARS/MW	Landrace	Cream
E47	MW3959	Andean	DARS/MW	Landrace	Light speckled
E48	MW365	Andean	DARS/MW	Landrace	Red Speckled
E50	MW3933	Andean	DARS/MW	Landrace	Red
E52	DRK57	Andean	DARS/MW	Landrace	Red
E56	MW3935	Andean	DARS/MW	Landrace	Dark red
E57	MW3971	Andean	DARS/MW	Landrace	Purple
E59	MW466	Andean	DARS/MW	Landrace	Red speckled
E60	MW3929B	Andean	DARS/MW	Landrace	Brown
E62	MW227	Andean	DARS/MW	Landrace	Yellow
E67	MW3921	Andean	DARS/MW	Landrace	Cream
E68	MW3929A	Andean	DARS/MW	Landrace	Cream
E70	MW3915	Andean	DARS/MW	Landrace	Dark red
E71	MW3917	Andean	DARS/MW	Landrace	Red mottled
E79	MW3934	Andean	DARS/MW	Landrace	Dark red
E81	MW3924	Mesoamerican	DARS/MW	Landrace	Brown
E82	MW3950	Andean	DARS/MW	Landrace	Cream
E84	MW3930	Mesoamerican	DARS/MW	Landrace	Cream
E90	MW3982	Andean	DARS/MW	Landrace	Red
E91	MW3945	Andean	DARS/MW	Landrace	Purple
E3	Nyambitira	Andean	DARS/MW	Released cultivar	Red
E4	VTTT924/4-4	Andean	CIAT	Released cultivar	Red speckled
E15	Nantupa	Andean	DARS/MW	Released cultivar	Red
E20	SCR64	Mesoamerican	CIAT	Released cultivar	Red
E27	NUA45	Andean	CIAT	Released cultivar	Red mottled
E39	SUGAR 131	Andean	DARS/MW	Released cultivar	Red speckled
E58	UBR(92)25	Mesoamerican	CIAT	Released cultivar	White
E63	NUA35	Andean	CIAT	Released cultivar	Red mottled
E80	VTTT924/10-4	Andean	CIAT	Released cultivar	Red
E85	NUA59	Andean	CIAT	Released cultivar	Red mottled
E92	SAA20	Andean	DARS/MW	Released cultivar	White

### Phenotyping trials

The 60 genotypes were evaluated in the field for agronomic performance. The genotypes were established at the Lilongwe University of Agriculture and Natural Resources (LUANAR), Bunda Horticulture Research farm (33.46°E and 13.10°S) in two years (2017/2018 and 2018/2019) during the main production seasons (November and April). The average rainfall per annum is 950 mm. The summer rainy season starts in November and ends in May. The site’s mean minimum and maximum annual temperatures were 16.5°C and 22.4°C, respectively. The site has dark loamy clay soils with soil pH of 5.8. The genotypes were planted in a 6 ×10 alpha lattice design with three replications. Each genotype was planted on a 3.00 m^2^ plot consisting of two 4m long rows. The spacing between row to row was 0.75 m, and between plant to plant was 0.10 m. Standard common bean cultivation practices were followed.

### Phenotypic data collection

Phenotypic data on qualitative and quantitative traits (Table 2) were collected following the International Board of Plant Genetic Resources [30]. The assessed qualitative traits were leaf shape (LS), flower colour (FC), growth habit (GH), leaf hairiness (LH), pod colour (PD), seed pattern (SP), seed colour (SC) and seed size (SS). Eleven quantitative traits were recorded: the number of nodes on the main stem from the base to first flower (NFF), recorded as a mean of five randomly selected plants per plot. The internode length from the first to the fifth node (FIL) was recorded as a mean length between the first and fifth nodes on the main stems of the five plants per plot. The width (WTL) and length (LTL) of the fifth trifoliate leaf were recorded as averages of trifoliate leaves measured on the sampled five plants. The days to 50 per cent flowering (DTF) were recorded as the number of days from the date of planting and to the date when 50 per cent of the plants in a plot had visible flowers, while the days to 90 percent maturity (DTM) were counted from the date of planting to the date when 90 per cent of the plants in a plot had reached physiological maturity. The number of pods per plant (NPP) was recorded as the average number of pods counted on five randomly selected plants at harvest. The number of seeds per pod (NSP) was recorded as the total number of seeds divided by the number of pods from five randomly selected plants at harvest. The seed length (SL) was recorded as the average length of five randomly selected seeds. Grain yield (GYD) was the weight of shelled grain harvested from all plants in a plot and converted to kilograms per hectare after adjusting for 12 per cent moisture content and according to plot size following [33].

$$GYD=\frac{10,000\;m^{2}}{Plot\;area(m^{2})}\times \frac{plot\;yield\;(g)}{1000\;g}\times \frac{100\;percent-MC}{100\;percent-12\;percent}$$

Where, GYD is grain yield per hectare in kilogram, and MC is the percentage moisture content of the grain at harvest. Hundred seed weight (HSW) was recorded as the weight of 100 randomly selected seeds after adjusting to 12 per cent moisture content.

**Table 2:** Agro-morphological traits used to characterise the common bean genotypes in the study.

Character	Abbreviation	Class /unit
Growth habit	GH	1 = type I (determinate-bush type), 2 = type II (indeterminate-bush type), 3 = type III (indeterminate-semi climber), 4 = type IV (indeterminate-climber)
Leaf shape	LS	1 = ovate, 2 = cordate, 3 = hastate, 4 = rhombohedral
Leaf hairiness	LH	1 = smooth, 2 = intermediate (moderately smooth), 3 = hairy
Length of trifoliate leaves	LTL	Cm
Number of nodes of first flower	NFF	Count
Day to 50per cent flowering	DTF	D
Width of trifoliate leaves	WTL	Cm
Fifth internode length	FIL	Cm
Flower colour	FC	1 = white, 2 = purple
Pod colour	PD	1 = green, 2 = brown stripes, 3 = red stripes, 4 = black strip
Day to 90per cent maturity	DTM	D
Number of pods/plant	NPP	Count
Number of seed/pod	NSP	Count
Seed length	SL	cm
Seed coat pattern	SP	1 = unpattern (single colour), 2 = pinto (painted or mottled), 3 = stripped (with colored strip lines), 4 = bicolor (with two colors only)
Seed size	SS	1 = large (>40 gram 100 seed weight), 2 = medium (25–40 grams 100 seed weight), 3 = small (<25 gram100 seed weight)
Seed coat colour	SC	1 = brown, 2 = cream, 3 = dark red, 4 = Khaki, 5 = Light speckled, 6 = Navy, 7 = purple, 8 = red, 9 = red mottled, 10 = red speckled, 11 = white, 12 = yellow.
Weight of 100 seed	HSWT	G
Grain yield	GYD	kg ha^-1^

cm = centimetres, g = grams, kg ha^-1^ = kilogram per hectare.

### Statistical analysis of phenotypic data

The frequency and significance tests of qualitative traits recorded among test genotypes were computed using the cross-tabulation procedure of SPSS version 26 [31]. Data on quantitative phenotypic traits were subjected to analysis of variance in GenStat 18^th^ edition [32]. Genotypes mean for quantitative traits were separated using the Fischer’s Unprotected Least Significant Difference at 5 per cent significance level. Further, multi-variate traits relationships among genotypes were deduced using the categorical principal component analysis (CATPCA) based on principal components (PC) with Eigen values above 1.00 in R software [33]. A communality value for each trait was calculated as the sum of squares of the PC loadings following [34] to identify well represented traits across the PCs.

### Genotyping

#### DNA extraction and genotyping

The 60 genotypes were profiled using SNP markers. The genotypes were planted in a greenhouse in seedlings trays and raised to the three-leaf stage. Genomic DNA was extracted from fresh leaves of the seedlings following the plant DNA extraction protocol of the Diversity Array Technology (DArT) [35]. After extraction, the DNA quality was checked for nucleic acid concentration and purity using a NanoDrop 2000 spectrophotometer (ND-2000 V3.5, NanoDrop Technologies Inc). The genomic DNA was shipped to Biosciences Eastern and Central Africa (BecA) hub of the International Livestock Research Institute (BecA-ILRI) in Nairobi, Kenya, for genotyping by sequencing. The DArTseq protocol was used to genotype samples using 17,190 silico DArT assigned to 11 chromosomes of the common bean. The quality of the SNP markers was determined by reproducibility and call rate [36]. The SNP markers used were of high quality with reproducibility values of 1.00, polymorphic information content (PIC) values ranging from 0.020 to 0.50, a mean call rate of 0.93 per cent ranging from 0.84 to 1.00. After eliminating the SNP markers with unknown chromosome positions and filtering markers with more than 10 per cent missing data, a total of 16 565 DArT silico were recovered and used in the analysis.

#### Genetic parameters and population structure analysis

Genomic data were imputed using the optimal imputation algorithm on the KDCompute server (https://kdcompute.igss-africa.org/kdcompute/). The polymorphic information content (PIC), minor allele frequency (MAF), observed heterozygosity (H_o_), genetic distance (GD), inbreeding coefficient (F_is_), and fixation index (F_st_) were estimated using the R package “adegenet” [37]. The population structure was determined by STRUCTURE2.3.4 software [38]. The length of the burn-in period and Markov Chain Monte Carlo (MCMC) were set at 10,000 iterations, and the model was run by varying the number of clusters (K) from 1 to 10 with 10 alterations for each K. The appropriate K value was estimated by implementing the Evanno method using the STRUCTURE Harvester program [39].

Analysis of molecular variance (AMOVA) and genetic diversity was performed using Power Marker V.3.25 [40] after grouping the accessions based on the gene pool and biological category as either landrace, breeding lines, or released varieties.

A joint analysis of phenotypic and genotypic data was conducted. A phenotypic distance matrix was generated based on Gower’s distance, while the genotypic distance matrix was generated using Jaccard’s coefficient. A combined matrix was developed from the summation of the genotypic and phenotypic matrices. The phenotypic, genotypic and combined matrices were used to generate hierarchical clusters using the package “cluster” in R software [43]. A comparison of the hierarchical clusters was conducted using the tanglegram function in “dendextend” package in R software [41].

## Results

### Phenotypic diversity and population structure analyses

#### Variation based on qualitative phenotypic traits

**Fig 1:**
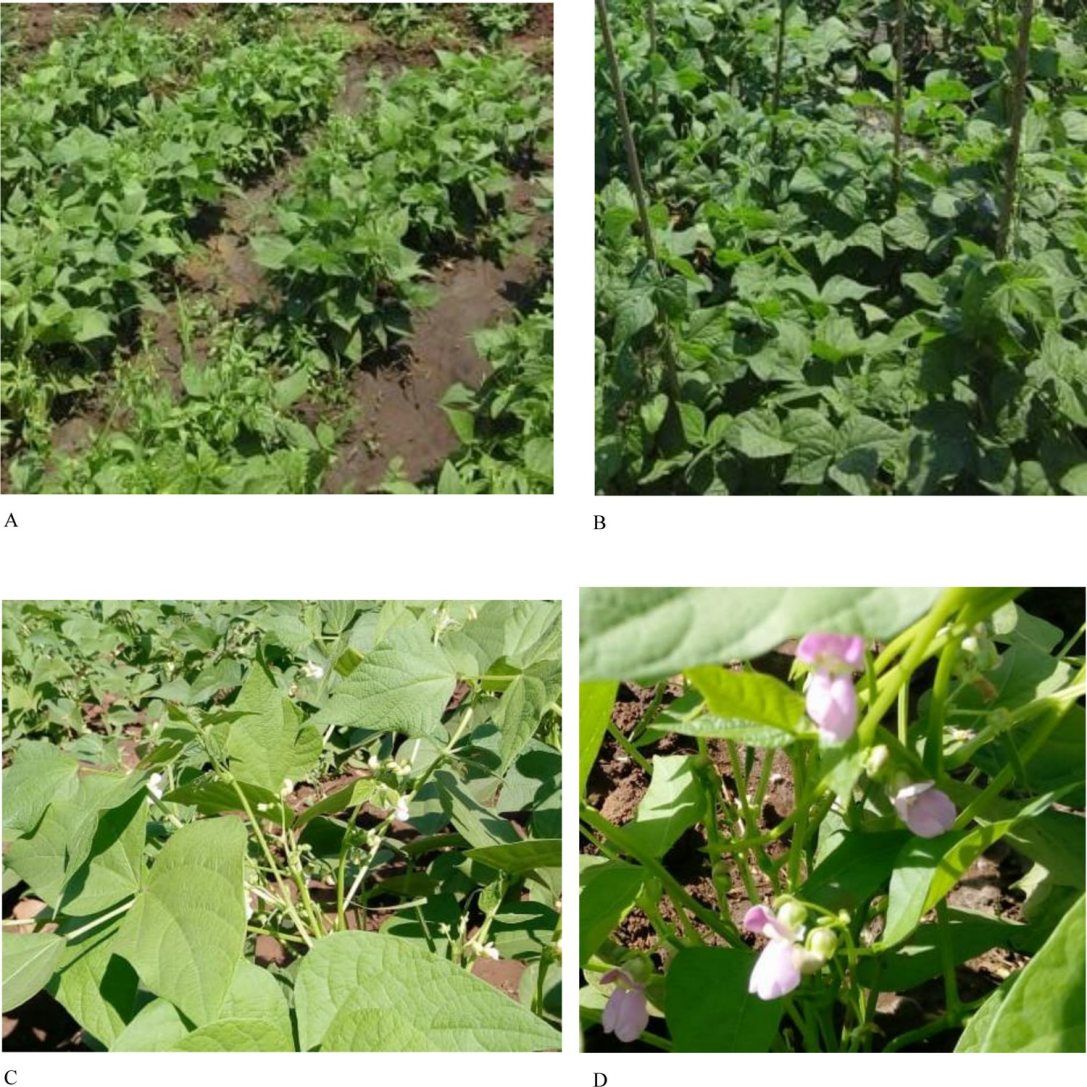
Growth type and flower colour among assessed common bean genotypes. Note: A–determinate growth type (genotype SUGAR 131), B–indeterminate growth type (MW3928), C–white flower (MW3969) and D–purple flower colour (MW227).

**Fig 2:**
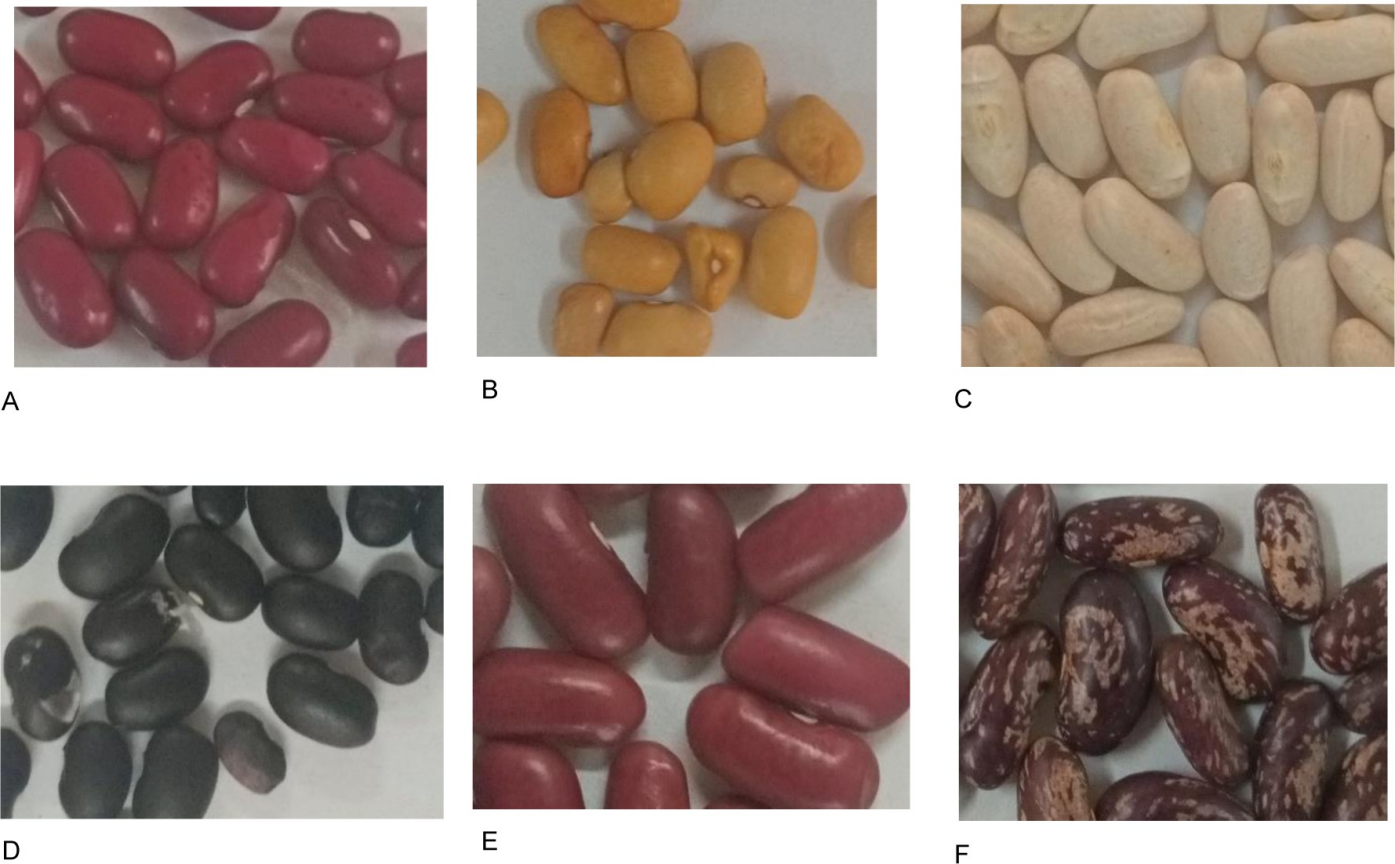
Variation in seed colour, size and shape among assessed bean genotypes. Note: A–Red, small, seed, kidney bean (SER265), B–Yellow, small seed,round bean, (MW3933) C–White, large seed, kidney bean (SAA20), D–Black,smallseed, kidney bean (genotype SEN125), E–Dark red, large seed, kidney bean (DRK57), F–Red mottled, large seed, kidney bean (NUA59).

The frequencies of eight qualitative traits and significant tests among the 60 test genotypes are presented in Table 3. Highly significant differences (p<0.001) were detected among the test genotypes for all assessed qualitative traits. The majority of the accessions (38 per cent) had ovate shaped leaves, while 32 per cent possessed cordate shaped leaves, 19 per cent hastate and 10 per cent had rhombohedral leaves. Additionally, 55 per cent of the test accessions had smooth-surface leaves, while 33 percent of the accessions had partially smooth leaves. Only 13 percent of the accessions had hairy leaves. The frequency of accessions with determinate growth habit was 35 percent. In contrast, the remainder of the accessions were indeterminate types that were further classified into three sub-groups: type II, III, and IV with relatively similar frequencies (Fig 1A and 1B). There were two main types of flower colour (Fig 1C and 1D). Fifty-nine percent of the test genotypes had white flowers, and 41percent had purple flowers. The test genotypes exhibited four distinct pod colours; green, red striped, black striped, and brown striped with respective frequencies of 77, 13, 6, and 5 percent. The tested accessions showed prominent variation in seed colour, size, and shape (Fig 2A–2F). There were a total of 11 seed colour types, while the seed classes consisted of the small, medium, and large seed sizes.

**Table 3:** Frequency distribution and signficance tests comparing 60 common bean genotype based on qualitative traits.

Trait	Description	Frequency (per cent)	DF	Chi-square	Genotype code^a^
Leaf shape	Cordate	32.30	177	8733.00***	E3, E11, E34, E40, E49, E50, E52, E56, E57, E63, E68, E74, E81, E85, E89, E91
	Hastate	19.10			E16, E60, E70, E92
	Ovate	38.20			E2, E10, E12, E15, E24, E28, E33, E35, E36, E44, E45, E47,E48, E51, E58, E67, E69, E71, E78, E79, E80, E84, E93
	Rhombohedral	10.30			E14, E21, E22, E26, E27, E30, E39, E46, E59, E82, E90
Leaf hairiness	Smooth	54.50	118	5822.00***	E4, E6, E10, E11, E21, E26, E27, E33,E35, E38, E40, E44, E47, E49, E52, E56, E59, E62, E68, E69, E70, E78, E80, E81, E82, E84, E85, E91
	Intermediate	32.90			E3, E12, E14, E15, E16, E30, E39, E48, E50, E51, E58, E60, E67, E71, E79, E89, E90, E93
	Hairy	12.50			E22, E23, E28, E34, E36, E45, E46, E57, E74
Flower colour	Purple	40.80	59	2911.00***	E3, E10, E12, E15, E21, E22, E24, E30, E33, E36, E44, E45, E45, E46, E47, E69, E79, E80, E82, E84
	White	59.20			E26, E27, E28, E35, E39, E40, E50, E51, E52, E57, E58, E59,E60, E62, E63, E67, E71, E72, E74
Growth habit	Type I	34.90	177	8733.00***	E6, E12, E14, E15, E21, E22, E26, E27, E33, E39, E40, E44, E46, E48, E49, E56, E58, E59, E71, E78, E89, E92
	Type II	19.40			E6, E12, E14, E15, E21, E22, E26, E27, E33, E39, E40, E44, E46, E48, E49, E56, E58, E59, E71, E78, E89, E92
	Type III	22.70			E16, E24, E36, E52, E57, E69, E74, E79, E80, E85, E90
	Type IV	22.90			E10, E11, E23, E28, E35, E38, E47, E50, E60, E62, E63, E67, E81, E91
Pod colour	Green	76.50	177	8733.00**	E2, E4, E6, E11, E14, E16, E21, E22, E23, E24, E26, E27, E28, E34,E35, E38, E39, E40, E45, E46, E48, E50, E51, E52, E56, E57,E58, E59, E60, E62, E63, E70, E74,E78, E80
	Brown strip	5.10			E67, E81
	Red strip	12.50			E3, E12, E15, E30, E33, E79
	Black strip	5.90			E10, E36, E47, E49, E68, E69, E84
Seed pattern	Unpattern	68.50	118	5828.00**	E2, E3, E6, E9, E10, E11, E14, E15, E16, E21, E23, E24, E29, E30, E34, E35, E38, E44, E45, E46, E50, E52, E57, E60, E62, E68, E70, E72, E74, E79, E80, E81, E82, E84, E89, E90, E91, E92
	Pinto	18.70			E27, E36, E40, E51, E63, E78, E85, E93, E71
	Bicolour	12.90			E26, E47, E22, E4, E39, E48, E49, E12, E59
Seed coat colour	Brown	4.80	649	32054.0***	E60, E81
	Cream	16.30			E2, E9, E21, E24, E26, E34, E38, E67, E68, E82, E84, E97
	Dark red	12.6			E3, E14, E15, E35, E44, E52, E56, E70, E79
	Khaki	0.50			E16
	Light speckled	3.30			E22, E26, E47
	Navy	2.50			E22
	Purple	6.20			E33, E57, E91
	Red	21.30			E6, E10, E11, E23, E28, E30, E45,E50, E74, E80, E89, E90, E92
	Red mottled	18.70			E27, E36, E40, E51, E63, E71, E78, E85, E93
	Red speckled	9.60			E4, E12, E39, E48, E49, E59, E69
	White	2.00			E58
	Yellow	2.10			E62
Seed size	Large	62.40	118	5828.0***	E2, E3, E4, E10, E11, E14, E15, E16, E23, E26, E27, E33, E34, E39, E40, E45, E47, E48, E51, E52, E56, E60,E63, E67, E69, E70, E78, E79, E82, E85, E90, E91, E93
	Medium	19.40			E9, E12, E24, E35, E38, E50, E57, E59, E62, E68, E71, E80,
	Small	18.20			E6, E21, E22, E28, E30, E44, E47, E58, E74, E81, E84, E89

DF = degrees of freedom^,^

*** significant at p<0.001.

^a^See Table 1 for code of the genotypes.

#### Variation based on quantitative phenotypic traits

The combined analysis of variance revealed the presence of significant genotype × year interaction effects (p<0.05) for LTL, DTF, SL, HSWT and GYD (Table 4). The main effects for genotype were significant (p<0.05) for all evaluated quantitative traits, while the year main effects were significant (P<0.05) for FIL, DTF, DTM, NPP, SL, HSWT and GYD. The means for the phenotypic traits for the 60 genotypes were summarised in Table 5. Genotype MW3955 (entry 11) had the highest FIL (27.5 cm) followed by MW3928 (E12) (22.5 cm) and MW3241 (E44) (22.0 cm). The mean NPP was significantly higher in 2017/2018 than in2018/2019. During the 2018/2019 season, the genotype MW3924 (E81) attained the highest mean NPP of 23 followed by genotype VTTT924/4-4 (E4) (21) and UBR(92)25 (E58)(20). There was marked genotypic variation for GYD in 2017/2018. Genotypes MW3915 (with a mean grain yield of 2756 kgha^-1^) and NUA59 (2706 kgha^-1^) were the highest yielding genotypes in 2018, while the genotypes VTTT924/4-4 (2094 kgha^-1^) and DRK57 (1989 kgha^-1^) were the top-performing genotypes in 2018/2019.

**Table 4:** Mean squares and signficant tests for 11 quantitative agronomic traits among 60 common bean assessed in two years.

Source of Variation	DF	LTL	WTL	FIL	NNF	DTF	DTM	NNP	NSP	LS	HSWT	GYD
Year	1	0.10	0.71	12932.57***	0.10	26044.01***	4723.38***	1281.93***	1.47	0.10*	255.02**	71022250.00***
Rep(Year)	4	25.43***	18.84***	103.74***	8.13***	15.65	53.71	80.29***	1.15	0.00	35.61	5256322.00***
Rep(Block)	15	8.36***	2.42***	40.24*	5.31***	57.15***	165.69**	34.22**	0.56*	0.92***	395.23***	1968734.00***
Genotype	59	3.51***	1.39*	23.84*	10.36***	71.96***	160.40***	45.76***	1.64	5.56***	435.53***	497228.00**
Genotype x Year	59	0.08***	0.51	26.74	0.19	21.18	34.17	13.83	0.42	0.02**	87.35***	400684.00*
Error	221	1.66	0.87	20.37	0.88	9.34	36.77	15.56	1.08	0.10	27.76	293800.00

DF = degree of freedom, Rep = replication, LTL = length of the fifth trifoliate leaf, WTL = width of the fifth trifoliate leaf, FIL = length between first node to fifth node of the main stem, NFF = number of nodes at first flower, DTF = days-to-50per cent flowering, DTM = days-to-physiological maturity, NPP = number of pods per plant, NSP = number of seeds per pod, SL = seed length, HSWT = hundred seed weight, GYD = grain yield, *, ** and *** are significance levels at p ≤ 0.05, ** p ≤ 0.01, *** p ≤ 0.001, in that order.

**Table 5:** Mean value for grain yield and agronomic traits of 60 common bean genotypes tested in two years showing the top 10 and bottom 5 ranked genotypes across years based on grain yield.

Genotypes	LTL		WTL		NFF		FIL		DTF		DTM		NPP		NSP		SL		HSWT		GYD ^(^Kg/ha)	
	2018	2019	2018	2019	2018	2019	2018	2019	2018	2019	2018	2019	2018	2019	2018	2019	2018	2019	2018	2019	2018	2019
E4	11.00	11.00	6.67	6.00	7.00	7.33	28.00	11.00	52.00	37.00	91.00	85.00	19.00	21.00	6.00	6.00	0.70	1.13	23.33	23.00	1750.00	2094.00
E52	9.00	8.00	6.00	6.00	8.33	8.33	29.00	11.00	52.00	44.00	97.00	100.00	18.00	15.00	5.00	5.00	0.90	1.13	27.00	25.00	2639.00	1989.00
E85	11.00	11.00	6.33	6.67	9.00	9.00	26.00	10.00	57.00	38.00	94.00	84.00	20.00	15.00	5.33	5.00	0.90	0.93	33.00	29.33	2706.00	1839.00
E58	9.00	9.00	5.33	6.33	8.33	8.33	26.00	8.00	47.00	45.00	85.00	93.00	22.00	20.00	5.33	5.00	0.60	0.50	23.00	23.33	1722.00	1683.00
E51	12.00	12.00	6.33	6.33	6.33	7.00	30.00	13.00	52.00	35.00	88.00	81.00	12.00	9.00	4.33	4.00	1.50	1.27	44.67	48.00	1900.00	1483.00
E81	10.00	9.00	6.00	6.33	9.67	9.67	25.00	9.00	53.00	45.00	89.00	91	26.00	23.00	4.00	5.00	0.40	0.53	21.67	24.33	922.00	1450.00
E80	11.00	11.00	6.00	6.67	7.67	7.33	20.00	12.00	58.00	41.00	91.00	84.00	14.00	12.00	5.33	5.00	0.60	0.67	25.67	34.33	1311.00	1356.00
E89	10.00	10.00	5.33	5.67	8.00	7.67	25.00	11.00	63.00	42.00	86.00	76.00	17.00	17.00	4.33	6.00	0.60	0.77	23.33	17.67	1694.00	121.00
E16	11.00	11.00	6.33	6.00	8.67	9.00	23.00	14.00	58.00	41.00	90.00	8.00	17.00	12.00	4.67	5.00	1.40	1.30	53.67	56.33	2317.00	1194.00
E40	13.00	13.00	7.00	7.00	5.67	5.33	25.00	15.00	49.00	33.00	85.00	79.00	14.00	10.00	4.33	5.00	1.30	1.23	44.00	57.33	2061.00	1144.00
E60	10.00	10.00	5.00	6.00	7.67	7.33	23.00	17.00	54.00	36.00	85.00	77.00	14.00	10.00	4.33	4.00	1.20	1.17	32.00	42.33	2011.00	472.00
E56	13.00	13.00	6.67	7.00	6.00	6.00	22.00	15.00	49.00	34.00	88.00	83.00	14.00	9.00	4.67	5.00	1.30	1.17	35.33	27.33	1944.00	411.00
E21	11.00	11.00	6.00	5.67	9.00	8.67	22.00	10.00	56.00	38.00	91.00	83.00	14.00	10.00	5,.00	5.00	0.80	0.70	32.67	33.33	1033.00	394.00
E68	10.00	10.00	6.33	7.00	9.00	8.33	22.00	11.00	59.00	39.00	98.00	90.00	15.00	9.00	3.67	4.00	0.90	1.10	27.67	26.33	1256.00	389.00
E59	10.00	10.00	5.33	5.33	5.33	5.33	24.00	12.00	53.00	33.00	90.00	81.00	21.00	12.00	4.67	4.00	1.20	1.03	44.00	42.67	1794.00	289.00
Mean	11.00	11.00	6.18	6.09	7.23	7.20	25.00	13.00	54.00	37.00	89.00	82.00	15.00	12.00	4.56	4.00	1.10	1.08	37.66	35.97	1781.00	893.00
Genotype_(0.05_ LSD)	2.10	2.20	1.60	1.59	1.56	1.68	9.20	5.10	5.80	4.20	7.70	11.70	6.30	6.60	0.15	1.50	0.29	0.27	10.86	5.99	1169.90	678.40
Year _(0.05_ LSD)	1.46		1.06		1.07		5.14		3.48		6.90		4.49		1.18		0,2		6.00		616.70	
CV%	5.50	6.40	10.30	7.80	4.60	5.60	7.00	4.50	1.30	0.70	1.00	1.20	6.40	11.30	3.30	2.80	2,9	1,1	2.80	0.60	21.50	18.90

LTL = length of the fifth trifoliate leaf (cm), WTL = width of the fifth trifoliate leaf, (cm) FIL = length between first to fifth node of the main stem (cm), NFF = number of nodes at first flower, DTF = days-to-50per cent flowering, DTM = days-to-physiological maturity, NPP = number of pods per plant, NSP = number of seed per pod, SL = seed length (cm), HSWT = hundred seed weight (g/100 seed), GYD = the grain yield (kg/ha).

^a^See Table 1 for code of the genotypes.

#### Principal component and bi-plot analyses based on phenotypic traits

Principal component (PC) analysis showed that the first seven PCs with Eigen values above 1.00 accounted for 74.10 percent of the total variation among the test genotypes (Table 6). The first and second principal components (PC1 and PC2, respectively) accounted for a total of 34.39 percent of the variation observed among the accessions. The traits with the highest contribution on PC1 were NPP (with PC loading of 0.89), NNF (0.75), DTF (0.67), GH (0.54), SS (0.45) and GYD (0.35). Traits, including LTL, SL and HSWT were negatively correlated with PC1. GYD (with PC loading of 0.65), FC (0.63), HSWT (0.31), FIL (0.30) and SC (0.12) were the highest contributors on PC2. Conversely, traits including PD, GH, DTF, and NNF exhibited moderate to strong negative loadings on PC2. All the traits exhibited generally high communalities above 0.58. However, NFF, PD, SC and DTF exhibited the highest communalities above 0.80.

**Table 6:** Eigen-values, proportion of variability and loading scores for the first seven PCs among 60 common bean genotypes evaluated in two years.

Traits	PC1	PC2	PC3	PC4	PC5	PC6	PC7	Communalities
Eigen values	3.94	2.25	1.76	1.7	1.39	1.29	1.02	
Proportion of variation (per cent)	21.91	12.48	9.75	9.42	7.73	7.15	5.67	
Cumulative variation (per cent)	21.91	34.39	44.14	53.55	61.29	68.43	74.1	
PD	-0.24	-0.76	-0.16	0.15	-0.19	0.24	0.22	0.83
DTF	0.70	-0.37	0.24	0.16	0.24	-0.03	-0.22	0.82
DTM	0.61	-0.11	0.14	0.28	-0.01	-0.11	0.30	0.58
FIL	-0.11	0.29	-0.26	-0.20	0.15	0.62	0.03	0.61
GYD	0.35	0.65	-0.01	0.30	-0.09	0.17	-0.13	0.69
HSWT	-0.40	0.31	0.54	0.01	0.28	0.26	0.02	0.69
LTL	-0.52	0.09	0.53	0.27	-0.30	0.06	-0.28	0.80
NFF	0.75	-0.36	0.34	0.07	0.02	0.13	-0.15	0.85
NPP	0.86	0.04	0.04	0.10	-0.12	0.17	0.04	0.80
NSP	0.37	0.10	-0.53	0.10	-0.19	0.25	-0.47	0.76
WTL	-0.21	0.06	0.38	0.66	-0.41	-0.01	-0.01	0.80
GH	0.54	-0.38	0.32	-0.17	0.20	0.25	-0.06	0.67
SS	0.45	0.27	-0.25	0.30	-0.04	0.36	0.34	0.67
SP	-0.34	-0.34	-0.21	0.50	0.28	0.07	0.38	0.75
SL	-0.52	-0.13	0.28	-0.01	0.32	0.56	-0.07	0.79
SC	0.28	0.12	0.38	-0.68	-0.20	0.01	0.30	0.83
LH	-0.02	-0.01	-0.10	0.20	0.72	-0.27	-0.25	0.70
FC	0.28	0.63	0.19	0.18	0.32	-0.18	0.27	0.75

PC = principal component, PD = pod colour, DTF = days-to-50percent flowering, DTM = days-to-physiological maturity, FIL = length between first node to fifth node of the main stem, GYD = grain yield, HSWT = hundred seed weight, LTL = length of fifth trifoliate leaves, NFF = number of nodes at first flower, NPP = number of pods per plant, NSP = number of seed per pod, WTL = width of the fifth trifoliate leaf, GH = growth habit, SS = seed size, SP = seed pattern, SL is the seed length, SC = seed colour, LH = leaf hairiness, FC = flower colour.

**Fig 3:**
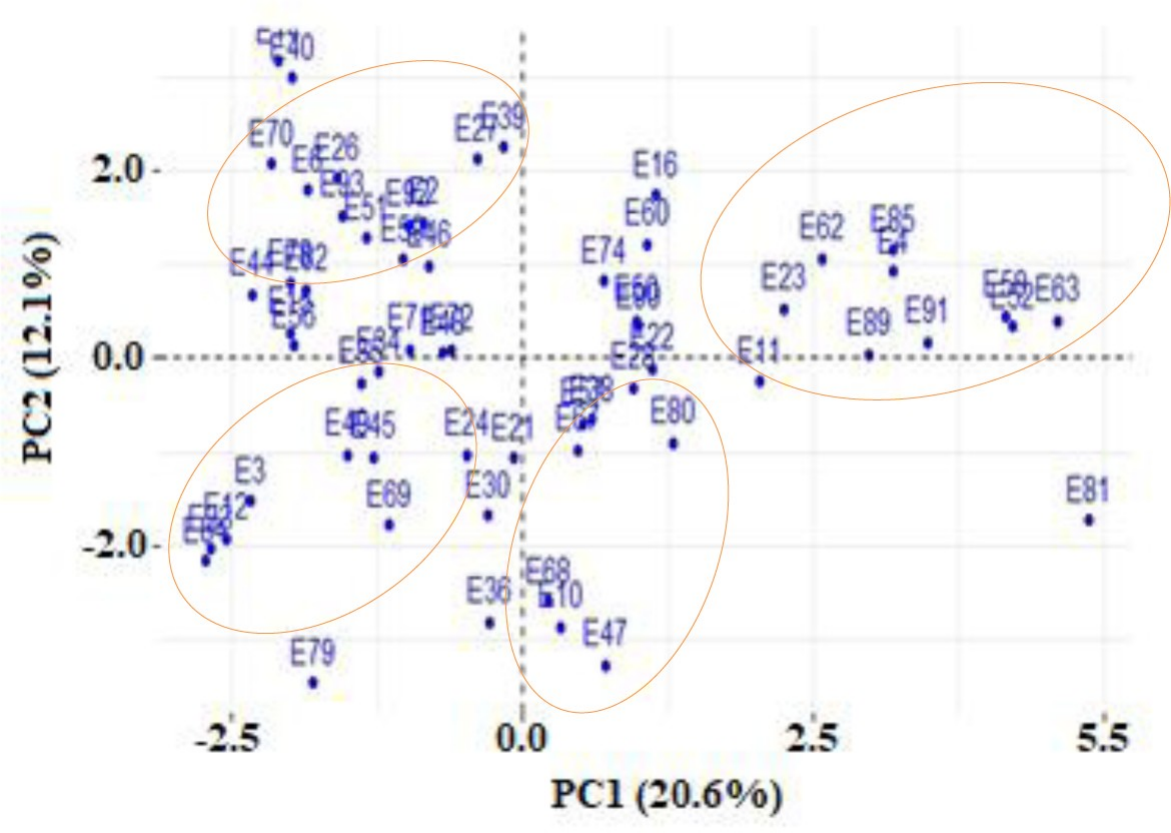
Principal commonent biplot of 60 common bean genotypes evaluated in two years.

The bi-plot clustered all genotypes into four groups (Fig 3). Genotypes in quadrant I were high yielding, followed by those in quadrant II. The low yielding genotypes were clustered in quadrant IV. Genotypes that were clustered in quadrant I include: angular leaf spot resistant (ALS) accessions such as A344 (E89), DRK57 (E52), NUA35 (E63), NUA59 (E85) and UBR(25)9 (E58). These genotypes are late maturing, high yielding and resistant to multiple stresses. MW3945 (E91), MW3955 (E11) and MW3933 (E50) were landraces that were grouped in quadrant I. VTTT924/10-4 (E80), a released large red kidney bean cultivar resistant to angular leaf spot (ALS) was found in quadrant II with landraces such as MW3924 (E81), MW3969 (E10) and MW3959 (E47). Quadrant III had two genotypes: Nyambitira (E3), a dark red kidney bean that is resistant to bruchids, and G11982 (E69), a genotype resistant to common bean mosaic virus (BCMV). Genotypes grouped in quadrant IV included CAL143 (E51), which is resistant to ALS, SUGAR131 (E39) that is resistant to BCMV, and Nantupa (E15) that is resistant to bruchids and NUA45 (E27), an early maturing and drought-tolerant genotype. Nyambitira and Nantupa were bred by DARS, Malawi, and the rest of the genotypes were developed at CIAT and released in Malawi in partnership with DARS, Malawi.

### Genetic diversity and population structure based on SNP markers

#### Population allelic diversity

The mean MAF was similar among Andean and Mesoamerican gene pools and breeding lines, landraces, and released varieties (Table 7). In addition, the SNP markers were moderately informative with a mean PIC value of 0.22, while the tested accessions were moderately heterozygous with a mean heterozygosity value of 0.45. The genotypes from the Mesoamerican gene exhibited higher heterozygosity (0.52) than the Andean genotypes (0.44). The varieties and landraces exhibited slightly higher than the breeding lines. The breeding lines exhibited the highest inbreeding coefficient of -0.68 compared to -0.60 exhibited by released varieties.

**Table 7:** Genetic parameters for different gene pools and types of genotypes of common bean based on 16,565 SNP markers.

Parameter	Overall	Andean	Mesoamerica	Breeding lines	Landraces	Released varieties
GD	0.28	0.26	0.32	0.26	0.28	0.3
PIC	0.22	0.20	0.25	0.20	0.22	0.24
MAF	0.24	0.23	0.26	0.22	0.24	0.24
Ho	0.45	0.44	0.52	0.42	0.46	0.47
F	-0.61	-0.68	-0.61	-0.63	-0.62	-0.6
Va	4628.7	4302.49	5238.15	4229.53	4607.4	4867.87
Vd	1940.24	1887.33	2244.52	1849.58	1941.96	2012.98

GD = Gene diversity, PIC = Polymorphic information content, MAF = Marker allelic Frequency, Ho = Observed heterozygosity, F = Fixation index, Va = additive variance, Vd = dominance variance.

#### Population structure

**Fig 4:**
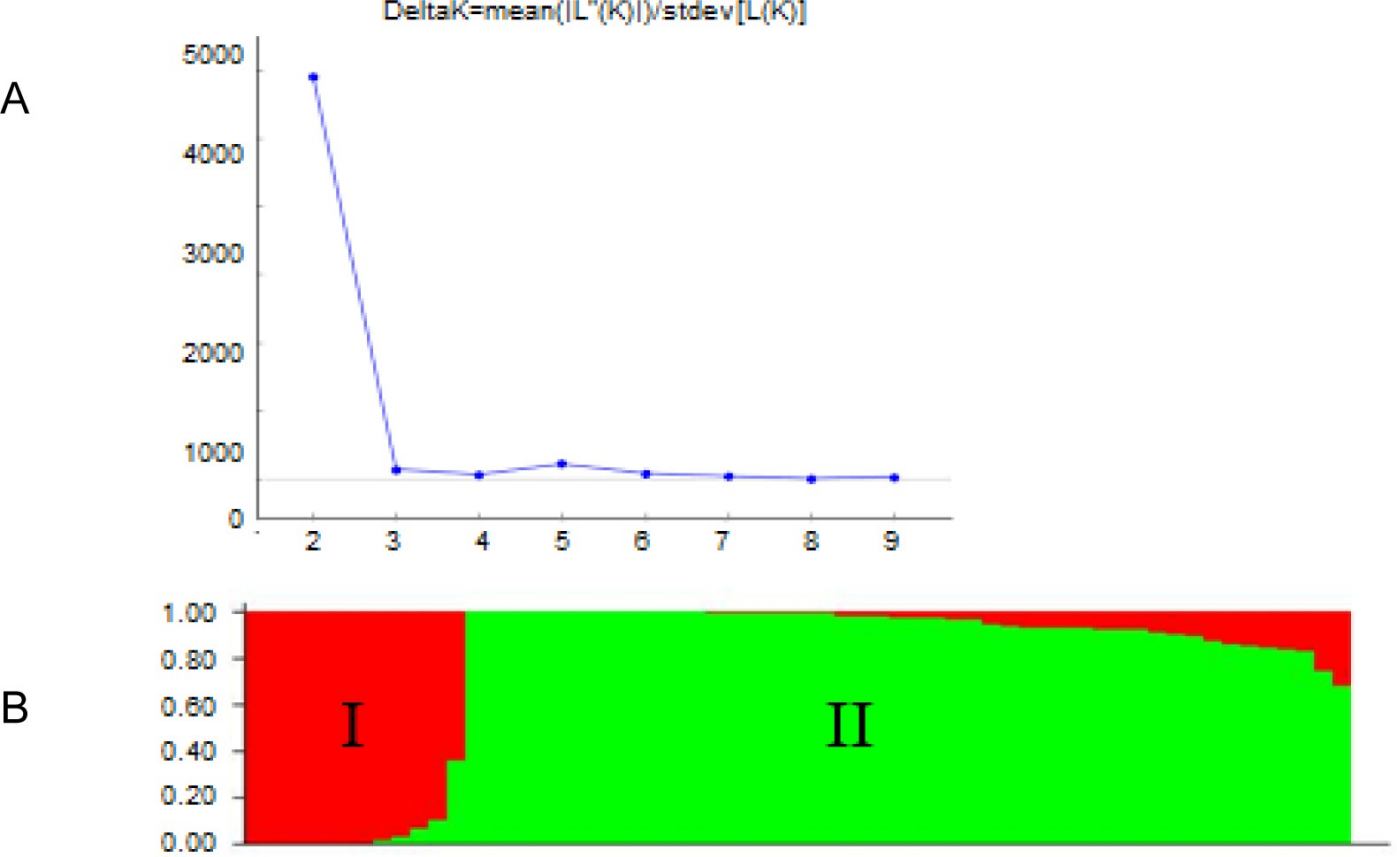
Population structure of 60common bean genotypes based on 16565 SNP markers. A. Highest delta K values showing K = 2. B. Genotype membership to the two clusters.

The population structure analysis delineated the 60 common bean genotypes into two groups based on the highest ΔK at K = 2 following the Evanno method (Fig 4A). The two identified groups were relatively similar in number (Fig 4B). Group I consisted of 52 percent of the test genotypes, which were mainly large-seeded. Group II had 48 percent of the test genotypes and comprised of the small-seeded bean types belonging to the Mesoamerican gene pool. Genotypes NUA45 (E27), NUA59 (E85), CAL143 (E51) and CAL96 (E78) belonging to the Andean gene pool, were clustered in Group I. The Mesoamerican types such as genotypes A222 (E76), A55 (E13) and A429 (E73) were grouped along with the small-seeded bean genotypes in Group II.

#### Cluster analysis

Cluster analysis based on SNPs markers grouped the 60 common bean genotypes into two main genetic groups (Table 8). Cluster I contained 50 genotypes, which was further divided into two sub-clusters (I-a and I-b). Sub-cluster I-a contained the genotype A429 (E73) only and I-b comprised of the rest of the genotypes. Similarly, Cluster II was divided into two sub-clusters: II-a and II-b. Sub-cluster II-a contained the genotype MW3960 (E36), and II-b comprised of the rest of the genotypes. Sub-clusters I-b and II-b were further divided into distinct sub-clusters based on origin, pedigree, morphology and agronomic performance. Genotypes NUA35 (E63), NUA59 (E59) and CAL96 (E78) and the breeding lines were clustered in the same sub-cluster II-b. NUA35 and NUA59 were derived from the backcross of CAL96/CAL96//G14519 for high iron and zinc content [42]. Additionally, Nantupa (E15) and Nyambitira (E3) NARS lines were clustered in the same sub-cluster as expected. These lines were half-sib families bred for bruchid resistance by DARS, Malawi. Nyambitira was derived from a cross of KK03 x KK25, and Nantupa derived from a Nagaga x KK25. Within sub-cluster I-b; breeding lines and SMC41 (E6), SMC104 (E86) and SMC166 (E31) were clustered in one sub-group. These lines were advanced backcross selections of SMC47/SN40//SCR1/SMC21. Conversely, NUA45 (E27) bred for high iron and zinc was found in sub-cluster II-b with its parental line CAL96 (E51).

**Table 8:** Clustering of 60 common bean genotypes based on 16,565 SNP markers.

Cluster	Entry code of genotypes^a^	FST	He
1	E30, E27, E28, E20, E40, E74, E58, E42, E81, E84, E89, E91	0.37	0.27
2	E2, E3, E4, E9, E10, E11, E12, E14, E15,E16, E21, E22, E23, E24, E26, E33, E34, E35, E36, E38, E39, E44, E44, E47, E48, E50, E52, E56, E57	0.61	0.13

Fst = Fixation index, He = Expected heterozygosity.

^a^See Table 1 for genotype codes.

#### Genetic differentiation among populations

The accessions were grouped into their gene pools, Mesoamerican or Andean, and their biological categories defined as breeding lines, landraces or released varieties. These were subjected to molecular analysis of variance. Results revealed that the variation within gene pools and among gene pools was significant (P<0.001) (Table 9). The variation between the gene pools accounted for 51 percent, while within the gene pool variance accounted for 49 percent of the total variation. Further, the variance was partitioned among breeding lines, landraces, and released varieties, showing no significant variation among the biological types. Within biological variance accounted for the total variation exhibited by the biological types. The extent of genetic differentiation (F_st_) among the biological categories ranged from -0.600 to -0.635 (Table 10). The highest F_st_ value was observed between landraces, while the lowest F_st_ value was between released varieties and breeding lines.

**Table 9:** Molecular analysis of variance of common bean populations based on 16,565 SNP markers.

Source	df	SS	MS	Est. Var.	% Variance
Among Gene pools	1	12044.26	12044.26	636.95	51%
Within Gene pools	58	34824.42	600.42	600.42	49%
Among Biological types	2	1003.39	501.70	0.00	0%
Within Biological types	57	45865.29	804.65	804.65	100%
Total	59	46868.68		804.65	100%

DF = degrees of freedom, E. variance = estimated variance, Gene pools = Andean or Mesoamerican, Biological types = breeding lines, landraces or released varieties.

**Table 10:** Population pair wise F_sts_ between populations of common bean genotypes.

Type	Breeding Lines	Landraces	Released Varieties
Breeding Lines	-	0.021	0.011
Landraces	-0.635	-	-0.008
Released Varieties	-0.600	-0.622	-

#### Combined analysis of phenotypic and genotypic data

**Fig 5:**
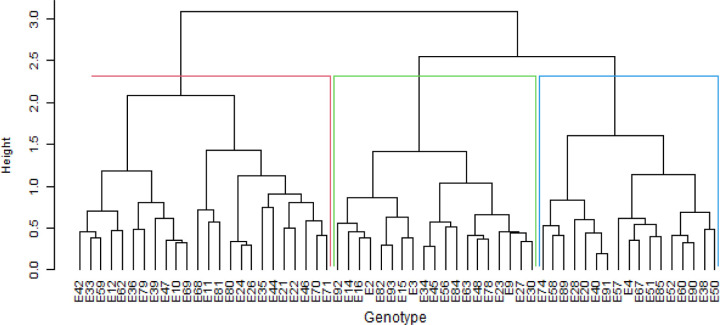
Dendrogram showing genetic relatedness among the 60 common bean genotypes based on the phenotypic matrix.

**Fig 6:**
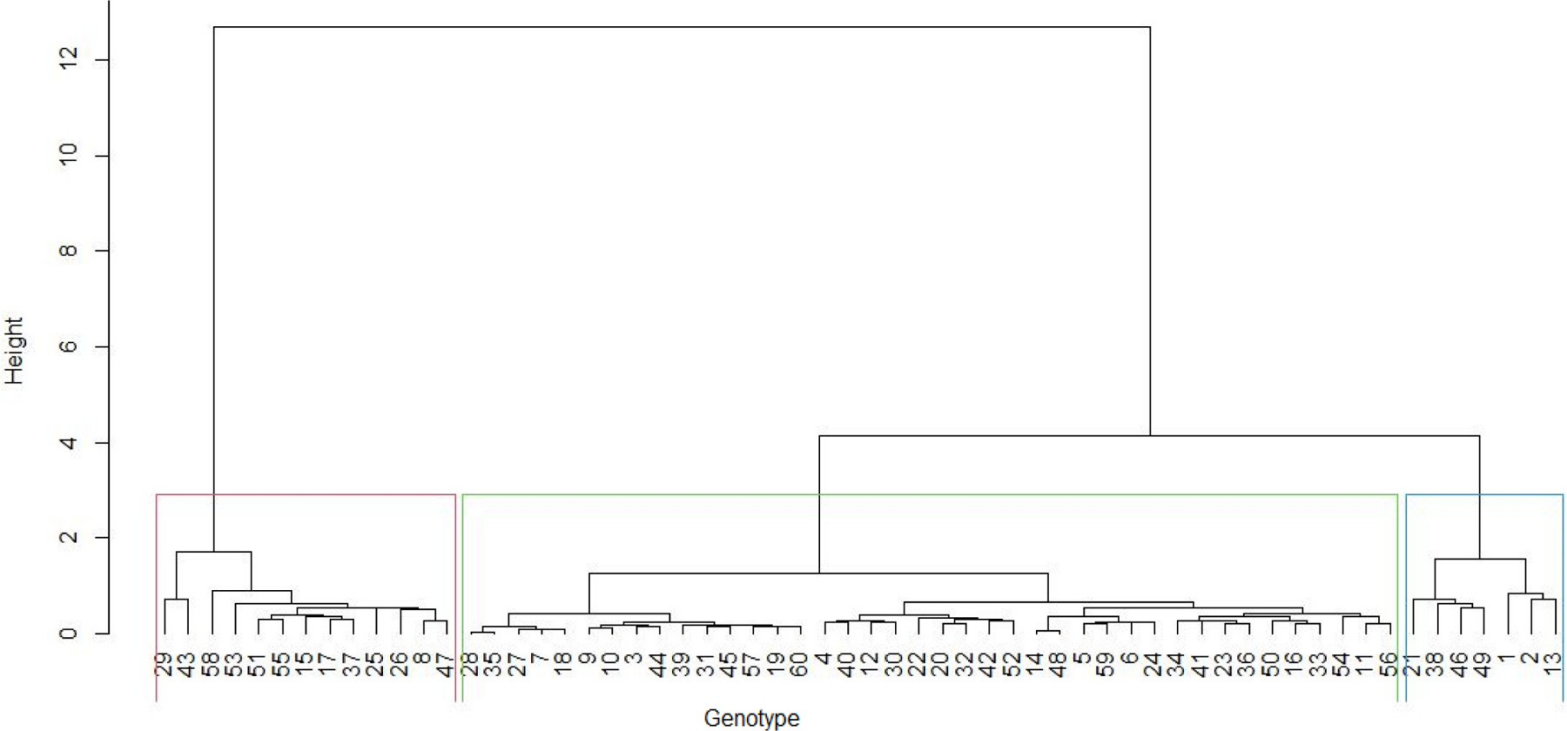
Dendrogram showing genetic relatedness among the 60 common bean genotypes based on the genotypic matrix.

**Fig 7:**
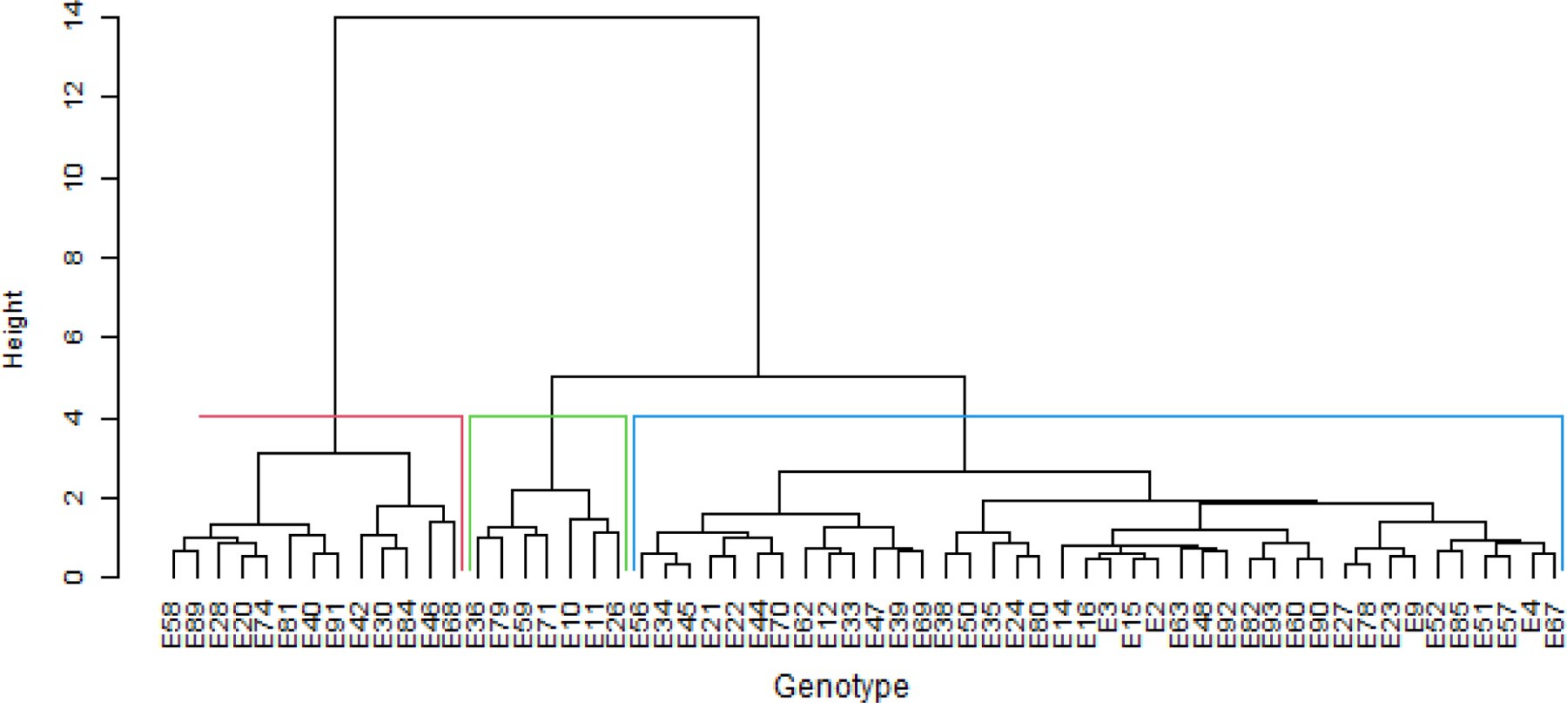
Dendrogram showing genetic relatedness among the 60 common bean genotypes based on the combined matrix.

**Fig 8:**
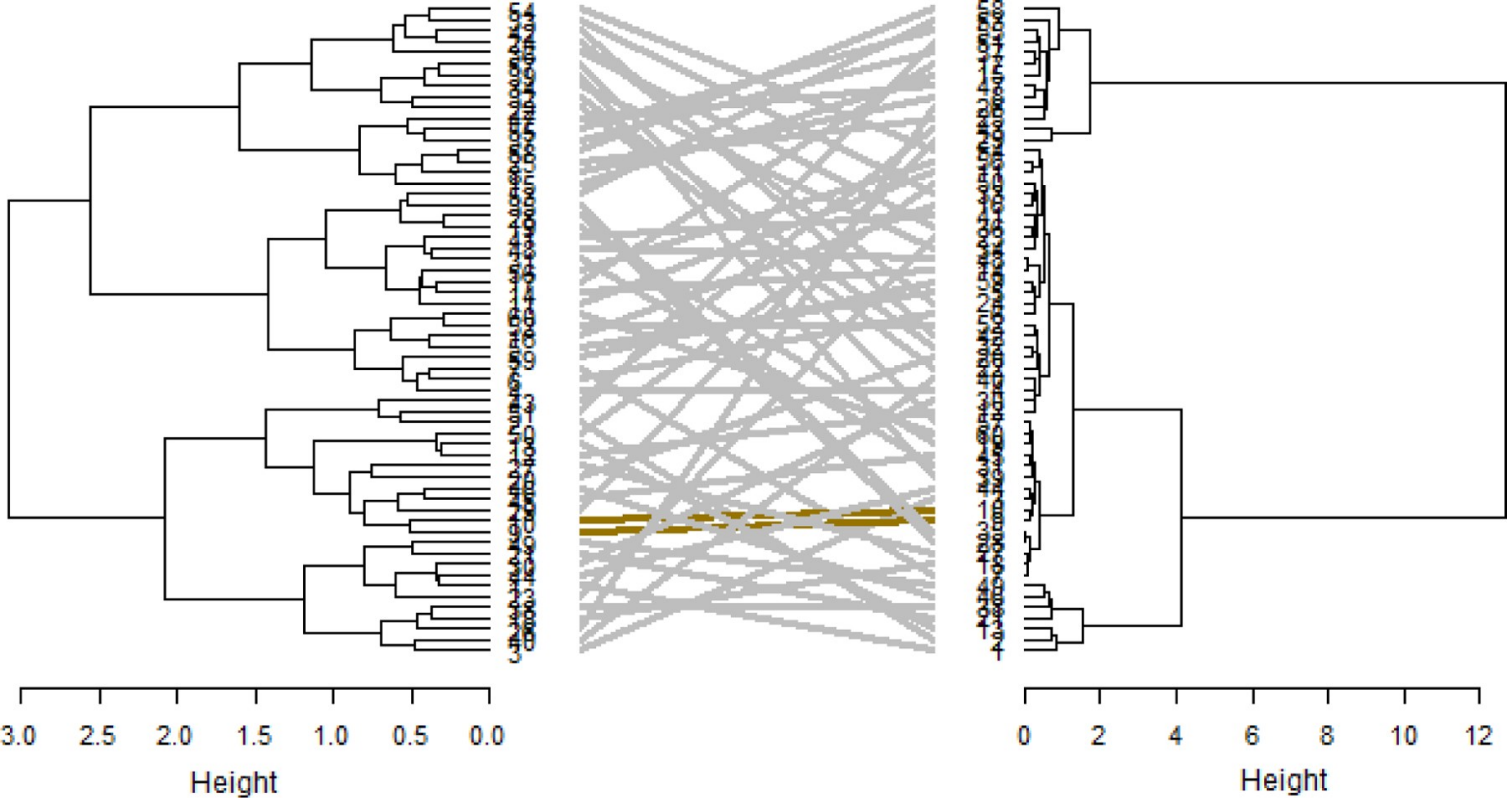
Tanglegram comparison of phenotypic and genotypic dendrograms.

The hierarchical clusters based on phenotypic and genotypic data revealed that the genotypes could be clustered into heterogeneous clusters. The phenotypic cluster showed that the first cluster was dominated by red seed coated Andean landraces obtained from Malawi (Fig 5). The second cluster comprised a mixture of landraces, varieties and breeding lines with red or cream coloured seeds. The cluster also included genotypes of mixed colours. The genotype cluster dendrogram grouped the genotypes into six heterogeneous clusters (Fig 6). The clusters were irrespective of sources of origin or colour of seed coat. The joint matrix revealed three different sized clusters among the genotypes (Fig 7). The largest cluster comprised Andean red seed coloured genotypes, while the smallest cluster was made up of Andean genotypes with red mottled seed colour. The tanglegram revealed that a considerable number of genotypes (about 40 per cent) maintained their positions in both the phenotypic and genotypic hierarchical clusters (Fig 8). Only two genotypes, E9 and E10 (MW3969) maintained their clusters and positions.

## Discussion

Significant genotypic variations were observed among the tested common bean genotypes across two testing seasons for quantitative traits such as DTF, DTM, NPP, HSWT and GYD (Table 4). This suggested that the test genotypes harbour a genetic diversity to select complementary lines for breeding purposes. Variation in phenotypic traits among genotypes reflects the underlying differences in their genetic constitution [43]. The panel consisted of genotypes from the Mesoamerican and Andean gene pools, which evolved under different selection pressures and environmental adaptation resulting in morphological and physiological differentiation. These landraces exhibit intrinsic genetic variation for key quality traits compared with accessions introduced from CIAT. The variation suggests that differential selection pressures impacted their evolution, resulting in genetic diversity observed among the landraces. The differential selection pressure is attributable to variability in climatic conditions, agronomic practices, natural selection and artificial selection by farmers over a long agricultural history. For instance, genotypes MW3915 (entry number E70), MW3966 (E40), MW3241 (E44) and MW3955 (E11) sourced from smallholder farmers from Malawi attained higher yields than CIAT genotypes such as SER124 (E74), A344 (E89), A286 (E28), SUGAR134 (E39) and NUA45 (E27). This may be attributed to the differences in genetic constitutions, adaptation to the climatic conditions, and local production practices in Malawi.

Qualitative traits such as growth habit, seed size and seed colour are important traits to farmers and consumers and are critical determinants for cultivar adoption [44]. For instance, a high frequency of accessions with smooth leaf types compared to non-smooth types suggests a long history of selection by farmers [45]. Farmers and consumers are also known to have preferences related to seed size, colour and shape. In Malawi, varieties with large seed sizes are preferred over varieties with medium and small-sized seeds. The most preferred seed coat colours in the country include red, red mottled and red speckled [46]. Traits such as seed coat colour, shape and size are usually controlled by a few major genes and present few challenges during selection [47]. In contrast, traits such as grain yield and maturity are polygenic and more difficult to improve by direct selection [48]. Therefore, to enhance varietal adoption among farmers, varietal development must incorporate both high grain yield potential and farmers-preferred quality traits through the recurrent selection for qualitative and quantitative traits [16, 49].

The differences in agronomic traits provide opportunities to select accessions that are suitable for diverse environments. The extent of genetic variation among genotypes in a breeding population or germplasm collections maintained at gene banks is a fundamental requirement for any crop improvement program [15]. For instance, farmers and breeders can select accessions with early maturity for environments with short rainy seasons as a mechanism to escape terminal drought stress. The significant variation in DTF and DTM observed among the accessions (Table 4) is important, especially for developing cultivars for drought-prone environments where early flowering and maturity contribute to drought escape. Earliness to flowering and maturity are desirable traits, especially in Southern Africa, where rainfall seasons are progressively becoming shorter due to climate change [16]. Long maturity type genotypes such as MW227 (E62), MW3945 (E91) and MW4012 (E22) from the DARS, Malawi gene bank and breeding lines such as DRK57 (E52), NUA35 (E63) from CIAT are useful genetic resources for long season rainfall environments.

The first two PCs (Table 6) revealed low morphological variation (34 per cent) among the evaluated genotypes, which suggest that there was a need for a higher number of components to discriminate the genotypes adequately. The inclusion of qualitative traits with discrete categories reduced the effectiveness of the PCs to explain the variation. In addition, the inclusion of breeding lines and commercial cultivars with a narrow range of genetic diversity also reduces the effectiveness of PCs [50]. Similarly, other studies have reported low variation for the first two principal components [14, 51]. The first two PCs explained only 33 percent of the total phenotypic variation in Brazilian common bean germplasm [51]. All the traits exhibited high communalities values across all the important PCs showing that the traits exhibited wide variation important in discriminating the genotypes. However, the study identified NFF, PD, SC and DTF as the most important descriptors based on their communalities values and will be useful for germplasm characterization and breeding. Genetic variation in GYD implies that superior genotypes with high GYD could be identified for developing breeding populations for common bean improvement using the test population.

The highest delta K value occurred at K = 2, which indicated that the 60 genotypes could be delineated into two sub-populations (Fig 4A). Similarly, the dendrogram clustered the accessions into two main clusters with two sub-clusters each (Table 8). The population structure analysis grouped the accessions into Andean and Mesoamerican gene pools in general. The results were consistent with previous reports on common bean, which reported these two major groups [36, 52]. The population structure also revealed that there were admixtures of common bean genotypes, which could be attributed to the inclusion of landraces in the study.

The Eastern and Southern Africa regions are recognized as centers of genetic diversity for common bean [11], and the germplasm adapted to these regions may no longer conform to the large Andean or Mesoamerican gene pools. In Malawi and most Eastern and Southern Africa countries, varietal mixtures in the common bean are common due to cropping practices, limited knowledge on the pedigree of bean types, and a lack of preference for varietal purity among consumers [21]. Varietal mixtures promote gene introgression through the natural crossing, thereby narrowing the genetic base [53]. The consequences of a narrow genetic base include low genetic gains and crop vulnerability to biotic and abiotic constraints [54]. The existence of admixtures requires fingerprinting to establish gene introgression and eliminate duplicate accessions to reduce the cost of germplasm management and facilitate the broadening of the genetic base in common bean.

Polymorphic information content values reveal the usefulness of particular markers in diversity studies [55]. In the present study, the mean PIC value was 0.22 (Table 7), which indicated that the SNP markers used were considered to be less to moderately informative. This could be due to the bi-allelic nature of SNP markers, which restrict PIC values to ≤ 0.5 [13, 56] and the low mutation rate of SNP markers [57]. Generally, SNP provides higher resolution in genetic studies, although they exhibit lower PIC values compared to other markers such as simple sequence repeats [58].

The mean observed heterozygosity (Table 7) in this study was 0.45, which was moderate and suggested that both recessive and dominant alleles were present in the germplasm. The similar heterozygosity values among the different types of genotypes showed that the genotypes contained both alternate alleles. The moderate heterozygosity also indicated that some of the accessions were possibly derived from uncontrolled outcrossing or were segregating at a number of loci. Common bean is naturally self-pollinating and would be expected to have lower heterozygosity estimates, as most loci would be homozygous [59]. It is important to have both recessive and dominant alleles expressed in a population to select adapted genotypes, although high expression of recessive alleles may drag selection efforts [60]. Variation in the magnitude of observed heterozygosity in common bean has been reported in several studies [26, 28, 61]. The differences could be attributed to the different germplasm used during evaluation. Previous studies on African common bean germplasm only considered landraces, while in the current study the test germplasm included breeding lines, landraces and varieties adapted to different ecologies.

Allele frequency information is useful in establishing the level of genetic differentiation in populations [62]. The low mean MAF of 0.24 found in this study for the whole population and low MAF values for breeding lines, landraces and varieties (Table 7) suggested a limited number of rare variants among the accessions, which indicate that the majority of genotypes shared common alleles. This implies that the successful use of the test population in a breeding program will depend on devising suitable selection strategies that can increase the expression of rare variants in the progeny and exploit their breeding value. Similarly, the mean MAF of 0.23 based on SNP markers was reported in Brazilian common bean core collection [38].

The low fixation index among the sub-populations in this study (Table 7) indicated low genetic variation among the populations and that the sub-populations were also genetically related. Fixation indices less than 0.05 indicate low genetic diversity between 0.05–0.15 moderate and greater than 0.15 indicate high divergence of genotypes [63]. In common bean, Fst values as low as -0.02 have been reported previously [64]. The main contributor to the high similarity among these populations is high gene introgression through artificial and natural outcrossing of common bean in improvement programs and farmers’ fields, respectively [8, 11]. The lowest fixation index recorded between breeding lines and landraces are concomitant to their shared ancestry. Breeding programs in Malawi often use the CIAT lines as breeding parents, and CIAT released most landraces cultivated in Malawi in partnership with DARS, Malawi. This is revealed by the low F_st_ between released varieties and the landraces.

The tanglegram comparing between phenotypic and genotypic clustering show that phenotypic and genotypic clusters were independent. The inconsistency between phenotypic and genotypic clusters is caused by environmental variance. Genotype × environment interaction confounds phenotypic performance, which reduces the correlation between genotype and phenotypic expression [65]. The genotypes used in this study consisted of diverse genotypes with different adaptation, which lead to deviation from their genetic potential. Inconsistencies between genotype and phenotype expressions have been reported previously in common beans (*Phaseolus* spp) [66]. A combined dendrogram based on genotypic and phenotypic data improves precision in genetic analyses of germplasm [67, 68]. The differential clustering of genotypes in the combined dendrogram showed that the combined dendrogram was independent of the phenotypic and genotypic matrices and can be used for more informative analysis [68].

## Conclusion

The present results showed that the test genotypes exhibited phenotypic variation under pinned by genetic diversity, which will facilitate selection and development of breeding populations for common bean improvement. The accessions exhibited a wide variation in traits such as FC, NNF, DTF, NPP, GH, DTM and GYD. Genetic analysis revealed that the accessions were divergent, although they could only be delineated into two populations clusters based on their origin. The variation between the clusters accounted for 51% while within cluster variation accounted for 49% of the total variation. The significant variation between the clusters was attributed to the differences in the evolution of Mesoamerican and the Andean gene pools. Improvement of common bean using this population would be achieved by developing breeding populations from crosses involving genetically divergent and superior parental lines of Mesoamerican origin such as SER124, A344 and UBR(92)25 crossed with Andean genotypes including DRK95, NUA59 and VTTT924/4-4. The narrow population structure and low genetic differentiation estimates showed that the genetic diversity in the present common bean germplasm should be harnessed by targeted crosses and new introductions to facilitate efficient selection and improvement. The discrepancy between genotypic and phenotypic analyses in identifying divergent genotypes highlighted that environmental variance was significant and measures to minimize its impact on selection should be employed.

## Supporting information

S1 File (XLSX)

S2 File (XLSX)
